# Supplementary material for: Burnout among intensivists and critical care fellows in South Korea: Current status and associated factors
Source: PLoS One. 2025 Feb 4;20(2):e0318495. doi: 10.1371/journal.pone.0318495 (PMC11793759; doi:10.1371/journal.pone.0318495)

Supplementary figure 1. Prevalence and severity of burnout among health care professionals. Percentage and number of (A) emotional exhaustion, (B) depersonalization, (C) personal accomplishment.


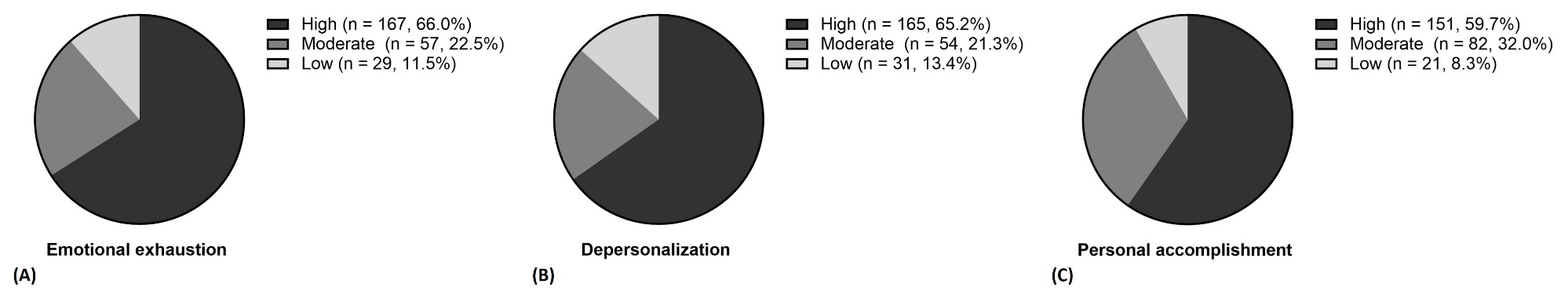

Supplement: S1 Fig — Percentage and number of (A) emotional exhaustion, (B) depersonalization, (C) personal accomplishment. (DOCX) [file pone.0318495.s001.docx]
