# Supplementary material for: Burnout among intensivists and critical care fellows in South Korea: Current status and associated factors
Source: PLoS One. 2025 Feb 4;20(2):e0318495. doi: 10.1371/journal.pone.0318495 (PMC11793759; doi:10.1371/journal.pone.0318495)
Supplement: Attachment — Submitted filename: Pone_comment.docx [file pone.0318495.s002.docx]

**Burnout among Intensivists and Critical Care Fellows in South Korea: Current Status**

**and Associated Factors**

Thank you for the opportunity to review a study. It is a topic widely studied in the nursing panorama but always offers insights into the phenomenon's evolution. My comments are below:

Introduction

The background seems well set, but we should expand the epidemiological part of the phenomenon to strengthen our reasons. For example, how widespread is burnout in nurses? And in critical care nurses? For this reason, I suggest reading this paper (DOI: 10.7429/pi.2022.751028), in which stratification by item is made on a national population. Furthermore, it is unclear what the authors mean by "Intensivists" and "Critical Care Fellows". I think these figures are present in the system where the data were collected, but how do we contextualize them internationally?

The passage on cultural aspects is interesting. It is often underestimated (in the text *- For example, values such as collectivism, perseverance, and hard work that are prevalent in many Asian cultures may have dual effects on burnout, potentially mitigating and exacerbating its development* [13, 14].)

The study aims to examine the associated factors. It is unclear whether these factors cause burnout or the intensive environment. Clarify this point.

Broadens the meaning of the study, why is it important?

Methods

I would write a separate paragraph titled "Ethical Considerations."

I need more information about data collection. Have response rates been calculated? Emailing the link often does not let us know if the person responding is someone we want to address. Be more explicit about data collection and write a separate paragraph.

Detailed information on the instrument used should be available. Likert scale? How many items per dimension? Psychometric properties? Is the instrument available in the language of the country where it was administered? I recommend using a checklist for reporting, e.g. STROBE in the methods.

Inclusion and exclusion criteria?

Describe the population and the risk factors more accurately. Why do we call them associated factors?

Results

The results are well presented; in Table 2, try inserting the p-value in the column for each factor.

Here, you define "Risk factors for burnout and severe burnout" as risk factors. Are you congruent throughout the manuscript regarding risk factors or associated factors?

Discussion

The discussions begin with the resumption of the objective and the declaration of the key points

the discussion offers a good overview of the phenomenon, but are we sure it is burnout? In the literature, a recent review explains the relationship between different phenomena, such as moral injury; I recommend reading it (DOI: 10.1177/09697330241281376). Furthermore, are there other phenomena that interact with burnout and abandonment? For example, regarding workplace violence, I suggest reading this interesting study (DOI: 10.7429/pi.2020.732089). What are the implications for the profession? For nursing managers? Broaden the discussion on preventive interventions.

Offer a unique perspective of this article to the magazine's readers.
