# Supplementary material for: Burnout among intensivists and critical care fellows in South Korea: Current status and associated factors
Source: PLoS One. 2025 Feb 4;20(2):e0318495. doi: 10.1371/journal.pone.0318495 (PMC11793759; doi:10.1371/journal.pone.0318495)
Supplement: Attachment — Submitted filename: Response to reviewer_20241216.docx [file pone.0318495.s003.docx]

Dear Editor

*Plos one*

Burnout among Intensivists and Critical Care Fellows in South Korea: Current Status and Associated Factors [PONE-D-24-43143]

I hope this letter finds you well. I am writing to submit the revised version of our manuscript titled "*Burnout among Intensivists and Critical Care Fellows in South Korea: Current Status and Associated Factors*", in response to the reviewers' comments and suggestions.

We have carefully considered the feedback provided by the reviewers and have made the necessary revisions to enhance the clarity and quality of our work. A detailed point-by-point response to the reviewers' comments is included with this submission. We have addressed each concern and suggestion comprehensively, and where appropriate, have revised the manuscript to reflect these changes.

We are grateful for the valuable feedback and believe that the revisions have strengthened the manuscript significantly. We hope that the revised version meets the standards of *Plos one* and will be considered favorably for publication.

Thank you again for granting us the additional time we requested. It would be an honor to have our work included in your esteemed publication. If you require any further information or additional changes, please do not hesitate to contact us.

Thank you for your time and consideration.

**<Reviewer 1>**

Thank you for the opportunity to review a study. It is a topic widely studied in the nursing panorama but always offers insights into the phenomenon's evolution. My comments are below:

Thank you for reviewing our study and providing your thoughtful comments. We appreciate your recognition of the topic's relevance to the field of nursing and its potential to provide valuable insights. We have carefully considered your feedback and made revisions accordingly.

**Introduction**

The background seems well set, but we should expand the epidemiological part of the phenomenon to strengthen our reasons. For example, how widespread is burnout in nurses? And in critical care nurses? For this reason, I suggest reading this paper (DOI: 10.7429/pi.2022.751028), in which stratification by item is made on a national population. Furthermore, it is unclear what the authors mean by "Intensivists" and "Critical Care Fellows". I think these figures are present in the system where the data were collected, but how do we contextualize them internationally?

Answer) Thank you very much for your valuable feedback. In response, we have added information from the referenced paper (DOI: 10.7429/pi.2022.751028) to the Background to better contextualize nurse burnout. In addition, we have clarified the definitions of "intensivists" and "critical care fellows" in the Methods section to improve understanding and ensure international relevance.

**Revised manuscript (Background)**

The COVID-19 pandemic has further exacerbated burnout, with one study [12] showing that more than half of nurses experienced emotional exhaustion and 85% experienced depersonalization.

**Revised manuscript (Methods)**

In this study, an "intensivist" was defined as a physician who has completed critical care training and is currently working in the ICU, and a "critical care fellow" was defined as a physician who is undergoing specialized training in critical care medicine and is actively working in the ICU. The inclusion criteria for this study were intensivists and critical care fellows who were actively working or training in ICUs at the time of the survey.

The passage on cultural aspects is interesting. It is often underestimated (in the text - For example, values such as collectivism, perseverance, and hard work that are prevalent in many Asian cultures may have dual effects on burnout, potentially mitigating and exacerbating its development [13, 14].)

The study aims to examine the associated factors. It is unclear whether these factors cause burnout or the intensive environment. Clarify this point.

Broadens the meaning of the study, why is it important?

Answer) Thank you for your insightful feedback. In response, we have revised the background section to explain how cultural values such as collectivism and perseverance can both mitigate and contribute to burnout, providing a clearer example of this dual effect. We have also addressed the diversity of healthcare systems and situations in Asia, explaining the importance of regional research to better understand burnout in this context, and re-emphasizing the need for research on burnout in each region due to these differences.

**Revised manuscript (Background)**

Despite these regional data, there is a lack of specific research focusing on South Korea. This gap is significant given the potential influence of cultural, economic, and systemic factors unique to South Korea. For example, cultural values such as collectivism and perseverance, which are prominent in South Korea, may promote social support but also increase vulnerability due to heightened expectations and self-imposed pressures [14, 15]. In addition, the diversity of health care systems and economic conditions across Asia limits the generalizability of findings from regional studies [16-18]. With its advanced healthcare system and unique workplace dynamics, South Korea warrants focused investigation to identify specific contributors to burnout and develop tailored strategies for prevention and intervention [19, 20].

**Methods**

I would write a separate paragraph titled "Ethical Considerations."

Answer) Thank you for your suggestion. We have added a separate section titled "Ethical Considerations" to clearly outline the ethical aspects of the study, ensuring greater transparency and alignment with ethical standards.

**Revised manuscript (Method)**

*Ethical considerations*

I need more information about data collection. Have response rates been calculated? Emailing the link often does not let us know if the person responding is someone we want to address. Be more explicit about data collection and write a separate paragraph.

Answer) Thank you for your valuable feedback and for highlighting the need for more detailed information on data collection. We have now provided additional details about the data collection process in a separate paragraph in the Methods section.

**Revised manuscript (Method)**

To encourage participation, the survey was announced on the Announcements section of the official website of the Korean Society of Critical Care Medicine (https://www.ksccm.org/), and additional efforts were made to engage participants by sending email and text message invitations directly to intensivists and critical care fellows. The survey responses were collected using NownSurvey (https://www.nownsurvey.com/), a proprietary online survey platform, and Google Forms, a publicly available web-based tool for the creation and management of surveys. Of the 502 individuals invited to participate, including intensivists and critical care fellows, 253 responded, resulting in a response rate of 50.4%.

Detailed information on the instrument used should be available. Likert scale? How many items per dimension? Psychometric properties? Is the instrument available in the language of the country where it was administered? I recommend using a checklist for reporting, e.g. STROBE in the methods.

Answer) Thank you very much for your valuable feedback. In response, we have included detailed information about the survey instrument used to measure burnout. The Maslach Burnout Inventory-Human Services Survey (MBI-HSS) was used, which consists of 22 items rated on a 7-point Likert scale ranging from "strongly disagree" to "strongly agree". These items assess three dimensions of burnout: emotional exhaustion, depersonalization, and personal accomplishment. The survey was administered in Korean, the native language of the participants, to ensure cultural and linguistic appropriateness. In addition, we confirmed that the psychometric properties of the Korean version of the MBI-HSS have been validated in previous studies. To increase transparency, we also referred to the STROBE checklist for reporting.

**Revised manuscript (Method)**

*Questionnaire*

Burnout was assessed using the Maslach Burnout Inventory-Human Services Survey (MBI-HSS) [2], a globally validated instrument consisting of 22 items divided into three domains: EE (9 items), DP (5 items), and PA (8 items). Each item was rated on a 7-point Likert scale ranging from "strongly disagree" to "strongly agree". EE scores were categorized as ≤ 18 (low), 19-26 (moderate), and ≥ 27 (high). DP scores were categorized as ≤ 5 (low), 6-9 (moderate), and ≥ 10 (high). PA scores were interpreted as ≥ 40 (low), 34-39 (moderate), and ≤ 33 (high).

The survey was administered in Korean to ensure cultural and linguistic relevance for participants, and the psychometric properties of the Korean version of the MBI-HSS have been validated in previous study [22].

Inclusion and exclusion criteria?

Describe the population and the risk factors more accurately. Why do we call them associated factors?

Answer) Thank you very much for your valuable feedback. We have clarified the inclusion and exclusion criteria in the Methods section. Specifically, the inclusion criteria were intensivists and critical care fellows who were actively working or training in ICUs at the time of the survey. The exclusion criteria were physicians not working in ICUs during the survey period. In addition, we have addressed your comment about "associated factors" by providing detailed information about the survey items in the Methods section.

**Revised manuscript (Method)**

The inclusion criteria for this study were intensivists and critical care fellows who were actively working or training in ICUs at the time of the survey. Physicians who were not actively working in ICUs during the survey period were excluded from participation.

**Results**

The results are well presented; in Table 2, try inserting the p-value in the column for each factor.

Answer) Thank you for your suggestion regarding Table 2. We have revised the table to include the p-value in the column for each factor as recommended.

**Revised manuscript (Table)**

Table 2. Demographic and Professional Characteristics of Participants

| **Characteristics** | **Total** | **Burnout** | **P-value** | **Severe burnout** | **P-value** |
| --- | --- | --- | --- | --- | --- |
| N | 253 | 223 |  | 150 |  |
| Age | 41.2 ± 7.6 | 40.8 ± 7.5 | 0.048 | 40.4 ± 6.5 | 0.069 |
|  |  |  |  |  |  |
| Sex |  |  | 0.070 |  | 0.636 |
| Male | 164 (64.8) | 149 (66.8) |  | 99 (66.0) |  |
| Female | 89 (35.2) | 74 (33.2) |  | 51 (34.0) |  |
| Position of intensivist |  |  | 0.011 |  | 0.102 |
| Professor | 59 (23.3) | 47 (21.1) |  | 28 (18.7) |  |
| Associate professor | 34 (13.4) | 28 (12.6) |  | 18 (12.0) |  |
| Assistant professor | 101 (39.9) | 90 (40.4) |  | 65 (43.3) |  |
| Fellow | 59 (23.3) | 58 (26.0) |  | 39 (26.0) |  |
| Marital status |  |  | 0.182 |  | 0.860 |
| Single, separated or widowed | 48 (19.0) | 45 (20.2) |  | 29 (19.3) |  |
| Married | 205 (81.0) | 178 (79.8) |  | 121 (80.7) |  |
| Religion | 167 (66.0) | 149 (66.8) | 0.459 | 107 (71.3) | 0.031 |
| Highest educational qualification |  |  | 0.069 |  | 0.258 |
| Bachelor’s degree | 29 (11.5) | 29 (13.0) |  | 19 (12.7) |  |
| Master’s degree | 107 (42.3) | 95 (42.6) |  | 68 (45.3) |  |
| Philosophy degree | 117 (46.2) | 99 (44.4) |  | 63 (42.0) |  |
| Monthly salary in USD |  |  | 0.307 |  | 0.435 |
| $3,083 – $4,624 | 57 (22.5) | 53 (23.8) |  | 30 (20.0) |  |
| $4,625 – $6,166 | 77 (30.4) | 69 (30.9) |  | 51 (34.0) |  |
| $6,167 – $7,707 | 77 (30.4) | 67 (30.0) |  | 44 (29.3) |  |
| Over $7,708 | 42 (16.6) | 34 (15.2) |  | 25 (16.7) |  |
| Length of service |  |  | 0.049 |  | 0.099 |
| 1 – 5 years | 144 (56.9) | 132 (59.2) |  | 88 (58.7) |  |
| 6 – 10 years | 63 (24.9) | 55 (24.7) |  | 41 (27.3) |  |
| > 10 years | 46 (18.2) | 36 (16.1) |  | 21 (14.0) |  |

Data are presented as mean ± standard deviation or number (%), unless otherwise indicated.

Here, you define "Risk factors for burnout and severe burnout" as risk factors. Are you congruent throughout the manuscript regarding risk factors or associated factors?

Answer) Thank you for pointing out the inconsistency in the use of the terms "risk factors" and "associated factors". We have carefully revised the manuscript to consistently use "associated factors" when describing statistical associations observed in the study.

**Revised manuscript (Results)**

*Factors associated with burnout and severe burnout*

**Discussion**

The discussions begin with the resumption of the objective and the declaration of the key points.

Answer) Thank you for your comments. We have revised the discussion to succinctly summarize the key findings and implications of the study, ensure alignment with the objectives, and highlight the critical factors associated with burnout.

**Revised manuscript (Discussion)**

This study reveals an alarmingly high prevalence of burnout among intensivists and critical care fellows in South Korea, with overall and severe burnout rates of 88.1% and 59.3%, respectively. The core components of burnout-emotional exhaustion, depersonalization, and reduced personal accomplishment-have significant implications for physician well-being, patient care, and the healthcare system. Key factors associated with burnout include job position, frequency of stay-home night calls, working in a medical ICU, and recent conflicts with colleagues. These findings highlight the need for targeted interventions that address both individual and systemic factors to mitigate burnout and support critical care physicians.

The discussion offers a good overview of the phenomenon, but are we sure it is burnout? In the literature, a recent review explains the relationship between different phenomena, such as moral injury; I recommend reading it (DOI: 10.1177/09697330241281376). Furthermore, are there other phenomena that interact with burnout and abandonment? For example, regarding workplace violence, I suggest reading this interesting study (DOI: 10.7429/pi.2020.732089). What are the implications for the profession? For nursing managers? Broaden the discussion on preventive interventions.

Answer) Thank you for your valuable comments and suggestions. We have discussed the interaction between burnout, moral injury, and workplace violence, reflecting the findings of the proposed papers. We have also added related content to the Discussion section.

**Revised manuscript (Discussion)**

In addition to burnout, other phenomena such as moral injury [38] and workplace violence [39] can exacerbate stress and emotional exhaustion. Moral injury, which results from situations that violate an individual's moral values, is strongly associated with anxiety, depression, and reduced quality of life. Similarly, workplace violence, including verbal and physical abuse, has been shown to undermine trust in management, increase anxiety, and amplify feelings of anger and distress. Addressing these interrelated factors requires implementing preventive measures, fostering ethical and respectful workplaces, and establishing reporting mechanisms to effectively manage incidents.

Offer a unique perspective of this article to the magazine's readers.

Answer) Thank you for your thoughtful comments and suggestions. To provide a unique perspective for the journal's readers, we have highlighted the novel aspects of our study, including the high prevalence of burnout among intensivists and critical care fellows in South Korea and the identification of associated factors such as job position, conflict with colleagues, and workload. In addition, we have contextualized these findings within broader phenomena such as moral injury and workplace violence, highlighting their interrelationships and implications for health care systems. We hope that this perspective will provide readers with valuable insights and stimulate further discussion on how to address burnout in critical care settings.

**<Reviewer #2>**

I would like to thank the authors for submitting a manuscript titled "Burnout among Intensivists and Critical Care Fellows in South Korea: Current Status and Associated Factors”.

This manuscript is based on a survey by the Korean Society of Critical Care Medicine to hospitals who provide CCM fellowship. Factors associated with burn out as defined by the Maslach Burnout Index are described. Below are specific comments and suggestions for improving the clarity, and educational content of the manuscript:

I would offer the following questions and/or suggestions.

Thank you for taking the time to review our manuscript and provide thoughtful comments and suggestions. We sincerely appreciate your detailed feedback, which will help us improve the clarity and educational value of our work. We will carefully consider each of your questions and suggestions to ensure that the manuscript meets the highest standards.

Please adjust the abstract to better reflect that this survey was done by the Korean Society of Critical Care Medicine, who the target was and what the purpose of the survey was. I cant see a response rate.

Answer) Thank you for your valuable comments. We have revised the abstract to clearly state that the survey was conducted by the Korean Society of Critical Care Medicine and to specify the target population and purpose of the survey. In addition, we have included the response rate to provide a complete overview.

**Revised manuscript (Abstract)**

This study, conducted by the Korean Society of Critical Care Medicine, aimed to investigate the prevalence and associated factors of burnout among intensivists and critical care fellows in South Korea.

Background: You write: Internationally, burnout rates among ICU professionals are alarmingly high, with studies reporting prevalence rates ranging from 33% to 47% in Western countries [9-11]. Delete the “however” at the start of the next sentence- it does not make sense. However, a recent cross-sectional survey of 159 ICUs in 16 Asian countries and regions found that the prevalence of burnout syndrome among ICU physicians and nurses in Asia accounts for 50-52% [12].

Background: You write: However, the prevalence and characteristics of burnout among ICU staff in Asian contexts, particularly in South Korea, remain understudied. Then you state: This research gap is significant given the potential impact of cultural, economic, and systemic factors on the experience of burnout. Obviously there is a study in Asian countries. Do you mean there is no data available in Korea and that is the gap? Please specify.

Answer) Thank you for your thoughtful feedback and for highlighting areas where the background section could be clarified. We have revised the text to address your concerns by explicitly stating that while there is data on burnout in Asia, specific research on ICU professionals in South Korea is lacking. We have also removed the unnecessary use of "however" to improve the flow of the text. In addition, we have emphasized the unique cultural, economic, and systemic factors in South Korea that may influence burnout, further justifying the need for this study. Thank you again for your valuable contributions.

**Revised manuscript (Background)**

Internationally, burnout rates among ICU professionals are alarmingly high, with studies reporting prevalence rates ranging from 33% to 47% in Western countries [9-11]. The COVID-19 pandemic has further exacerbated burnout, with one study [12] showing that more than half of nurses experienced emotional exhaustion and 85% experienced depersonalization. These findings highlight the critical need for targeted interventions for ICU staff. In Asia, a recent cross-sectional survey of 159 ICUs in 16 countries and regions reported a burnout prevalence of 50-52% among ICU physicians and nurses [13]. Despite these regional data, there is a lack of specific research focusing on South Korea. This gap is significant given the potential influence of cultural, economic, and systemic factors unique to South Korea. For example, cultural values such as collectivism and perseverance, which are prominent in South Korea, may promote social support but also increase vulnerability due to heightened expectations and self-imposed pressures [14, 15]. In addition, the diversity of health care systems and economic conditions across Asia limits the generalizability of findings from regional studies [16-18]. With its advanced healthcare system and unique workplace dynamics, South Korea warrants focused investigation to identify specific contributors to burnout and develop tailored strategies for prevention and intervention [19, 20].

Background: You write: Recognizing the high prevalence of burnout among medical professionals in various specialties, this study specifically aims to address the less explored area of burnout among those at the forefront of critical care in South Korea. However, you just previously mentioned studies looking at critical care clinicians, this is confusing. Please rewrite.

Answer) Thank you for pointing out the potential confusion in the background section. We have revised the text to clarify that while previous studies have examined burnout among critical care clinicians in other contexts, data specific to intensivists and critical care fellows in South Korea remain limited. The revised version highlights this research gap and the unique challenges faced by ICU staff in South Korea, ensuring that the focus of the study is clear.

**Revised manuscript (Background)**

Given the prevalence of burnout among medical professionals, this study focuses specifically on the unique challenges faced by intensivists and critical care fellows in South Korea. While previous studies have examined burnout among intensivists in other contexts, data specific to South Korea remain limited. The South Korean healthcare system is currently under significant pressure [21] , with ongoing medical disputes adding to the stress and workload of critical care clinicians.

Methods: The study design is unclear. Who sent out the survey. How did you know who to sent it to? Did you sent it to individuals or to institutions? You mention previous surveys. Needs to be rewritten.

Answer) Thank you very much for your valuable feedback. We have revised the Methods section to clarify the study design and address concerns. In particular, we have detailed that the Korean Society of Critical Care Medicine distributed the questionnaire to intensivists and critical care fellows working in the ICUs of hospitals that offer subspecialty training in critical care medicine. The society identified potential participants through its institutional and individual member databases. The survey was sent directly to individuals via email and text message to ensure that the target population was reached. We have included these details in the manuscript.

**Revised manuscript (Methods)**

To encourage participation, the survey was announced on the Announcements section of the official website of the Korean Society of Critical Care Medicine (https://www.ksccm.org/), and additional efforts were made to engage participants by sending email and text message invitations directly to intensivists and critical care fellows. The survey responses were collected using NownSurvey (https://www.nownsurvey.com/), a proprietary online survey platform, and Google Forms, a publicly available web-based tool for the creation and management of surveys.

Is the study IRB exempt or not. You mention an ethics approval

Answer) Thank you for your comment. We have clarified in the Methods section that the study was exempt from Institutional Review Board (IRB) review after an exemption request was submitted to the Chungnam National University Hospital IRB (No. 2023-11-036).

NownSurvey and Google Forms need to be referenced. I could not find NownSurvey online.

Answer) Thank you for your comment. We have revised the text in the Methods section to clarify that NownSurvey is a proprietary online survey platform and to provide additional context for Google Forms as a publicly available survey tool.

**Revised manuscript (Methods)**

To encourage participation, the survey was announced on the Announcements section of the official website of the Korean Society of Critical Care Medicine (https://www.ksccm.org/), and additional efforts were made to engage participants by sending email and text message invitations directly to intensivists and critical care fellows. The survey responses were collected using NownSurvey (https://www.nownsurvey.com/), a proprietary online survey platform, and Google Forms, a publicly available web-based tool for the creation and management of surveys.

Results: Whats the response rate?

Answer) Thank you for your comments. We have clarified the response rate in the results section to indicate that responses were received from 194 intensivists and 59 critical care fellows, for an overall response rate of 50.4%.

**Revised manuscript (Results)**

Responses were received from 194 intensivists and 59 critical care fellows actively working in these ICUs, for an overall response rate of 50.4%.

This is unclear: The position of the intensivist, especially for those at the level of assistant professor or fellow, was associated with a higher likelihood of burnout (OR, 3.916; 95% CI, 1.485 - 10.327; p = 0.006). All surveyed people were intensivists, right?

Answer) Thank you for your comment. We have revised the sentence to clarify and avoid ambiguity regarding the subgroups analyzed.

**Revised manuscript (Results)**

Among participants, those at the assistant professor or fellow level were associated with a higher likelihood of burnout (OR, 2.279; 95% CI, 1.223 - 4.247; p = 0.010).

Please delete the double brackets [[: Another study in France found the prevalence of burnout among intensivists to be approximately 32% [[16], further highlighting the disparity between regions. A similar survey from Asian ICUs found a burnout rate of 51% within the same professional cohort [[12].

Answer) Thank you for pointing out the problem with the double brackets. We have corrected this formatting error in the manuscript.

In this study 47.5 % of french intenivists had burnout: Embriaco N, Azoulay E, Barrau K, et al. High level of burnout in intensivists: prevalence and associated factors. Am J Respir Crit Care Med. 2007;175:686–92.

Studies have shown female gender is associated with higher rates of burnout- see study above or (Moll V, Meissen H, Pappas S, Xu K, Rimawi R, Buchman TG, Fisher L, Bakshi V, Zellinger M, Coopersmith CM. The Coronavirus Disease 2019 Pandemic Impacts Burnout Syndrome Differently Among Multiprofessional Critical Care Clinicians-A Longitudinal Survey Study. Crit Care Med. 2022 Mar 1;50(3):440-448.)

The discussion needs to be rewritten in a more concise manner.

Answer) Thank you for your valuable feedback. We have revised the Discussion section to be more concise and to integrate the results of the referenced studies.

**Revised manuscript (Discussion & limitation)**

In addition, this study was conducted using data collected before the COVID-19 pandemic. Other study [54] have shown that the pandemic significantly increased burnout rates, particularly among ICU nurses and female clinicians. For example, research [55] conducted during the pandemic in Korea reported that 77.3% of healthcare workers responding to the COVID-19 outbreak met criteria for burnout, with higher rates observed among females, younger individuals, and those with chronic fatigue or physical symptoms due to excessive workload.

How can the highest qualification be only a bachelors degree in 22.5% if all participants were physicians?

Answer) Thanks for your insightful comment. In South Korea, individuals become licensed physicians after graduating from medical school with a bachelor's degree in medicine. While some physicians pursue additional graduate degrees (master's or doctoral) during their internship, residency, or fellowship training, many do not, especially those in training or early in their careers. Because this study included fellows as participants, it is possible that 22.5% of respondents had only a bachelor's degree. We hope this clarifies the educational background of the participants.

How can the Stay-home night calls per month be associated with burnout but not the in house nights?

Answer) Thanks for your insightful comment. The association of home-based night call with burnout, but not that of in-house night call, may be due to several factors. Stay-home night calls blur the boundaries between personal and professional life because physicians remain on call at home, resulting in constant mental alertness and interrupted rest. Unlike hospital overnights, where controlled environments for rest are often available, stay-home calls may result in poorer sleep quality due to frequent interruptions, further exacerbating stress and emotional exhaustion. In addition, stay-home calls may be perceived as more intrusive because they extend work responsibilities into personal time, making it difficult for physicians to fully disconnect from their work. The unpredictability of being called during stay-home nights may also create anxiety that is less pronounced during the structured workload of in-house nights. In addition, the relative isolation of stay-home calls compared to the camaraderie and immediate support available during in-house nights may further increase feelings of stress and burnout. We hope this explanation provides clarity and addresses your concerns.

It is unclear if this survey only went to medical ICUs? There are no surgical, neuro, CVICU, etc?

Answer) Thank you for your comment. We have clarified this point in the manuscript. Table 1 provides details on the types of ICUs studied. Specifically, medical ICUs comprised 61.7% of the total, surgical ICUs 35.2%, and mixed ICUs 3.2%. Medical ICUs were defined as units that primarily admitted patients from internal medicine specialties, including neurology and general medicine. Surgical ICUs were defined as units admitting patients from surgical specialties, such as general surgery, neurosurgery, and cardiothoracic surgery. Mixed ICUs were defined as units caring for a combination of these patient populations.

The major issue though that I see is that the survey was done almost 6 years ago. Much has happened since -COVID! I would encourage the authors to repeat the survey now, in the post COVID world. The results might be different (worse?) now.

Answer) Thank you for your valuable comments. We have addressed your concerns by including additional details in the Limitations section of the manuscript, emphasizing that the survey was conducted prior to the COVID-19 pandemic. We acknowledged that the pandemic likely had a significant impact on burnout rates among critical care nurses and emphasized the importance of conducting follow-up studies in the post-COVID era to assess how these rates and contributing factors may have evolved. We appreciate your suggestion, which helped us to improve the clarity and relevance of the limitations of our study.

**Revised manuscript (Limitation)**

Our study has several limitations. First, although our findings provide valuable insights, they may not fully represent all ICUs in South Korea, and our results may be subject to response bias. The cross-sectional nature of this study limits the ability to draw causal inferences. In addition, despite an adequate participation rate, there is a potential for selection bias, as individuals experiencing burnout or depressive symptoms may have been more likely to respond, potentially inflating the prevalence rates. The use of the MBI, while globally accepted, may also be interpreted differently in different cultural and institutional contexts, which could influence the results. In addition, this study was conducted using data collected before the COVID-19 pandemic. Other study [54] have shown that the pandemic significantly increased burnout rates, particularly among ICU nurses and female clinicians. For example, research [55] conducted during the pandemic in Korea reported that 77.3% of healthcare workers responding to the COVID-19 outbreak met criteria for burnout, with higher rates observed among females, younger individuals, and those with chronic fatigue or physical symptoms due to excessive workload. Because our study does not capture the additional burden of the pandemic, future research is needed to assess the long-term impact of COVID-19 on burnout among critical care professionals in South Korea. Despite these limitations, our findings contribute to a deeper understanding of burnout among critical care professionals in South Korea and highlight the need for targeted interventions and further research to address this growing problem.
